# Nanomaterial-Based Sensing Systems to Detect Neuropharmaceutical Compounds and Neurotransmitters

**DOI:** 10.3390/s25113256

**Published:** 2025-05-22

**Authors:** Monireh Bakhshpour-Yücel, Nawal Aljayyousi, Bilgen Osman, Nese Lortlar Ünlü, Adil Denizli, M. Selim Ünlü

**Affiliations:** 1 Department of Chemistry, Faculty of Science and Art, Bursa Uludag University, Bursa 16059, Turkeynawal98yazid@gmail.com (N.A.); bilgeno@uludag.edu.tr (B.O.); 2 Faculty of Medicine, Histology and Embryology, Atlas University, İstanbul 34403, Turkey; selim@bu.edu; 3 Photonics Center, Department of Biomedical Engineering, Boston University, Boston, MA 02215, USA; 4 Department of Chemistry, Hacettepe University, Ankara 06800, Turkey; denizli@hacettepe.edu.tr; 5 Photonics Center, Department of Electrical Engineering, Boston University, Boston, MA 02215, USA

**Keywords:** nanomaterial, sensor systems, neuropharmaceutical, neurotransmitters

## Abstract

This review explores the application of nanomaterial-based sensing systems for precisely detecting neuropharmaceutical compounds and neurotransmitters, delving into the connections between nanotechnology and neuropharmacology. Nanotechnology appears as a promising solution for many significant challenges posed by the complexities of the brain’s biochemical nature. Using nanoscale materials, scientists have created novel sensors with high selectivity, sensitivity, and adaptability. Developing neuropharmaceutical compounds and monitoring their side effects on our neurological system raised the need for these nanomaterial-based sensors. In this review, we demonstrate the effectiveness of these technologies in real-time neuroactive compound detection and monitoring by illuminating the underlying principles through an examination of significant studies and recent developments. This review also highlights collaborative efforts at the intersection of nanotechnology and neuropharmacology and their direct and indirect effects on the understanding and controlling several neurological disorders. This review covers both sensors under research and those already applied in vivo or clinical monitoring of drug side effects.

## 1. Introduction

**Figure 1:**
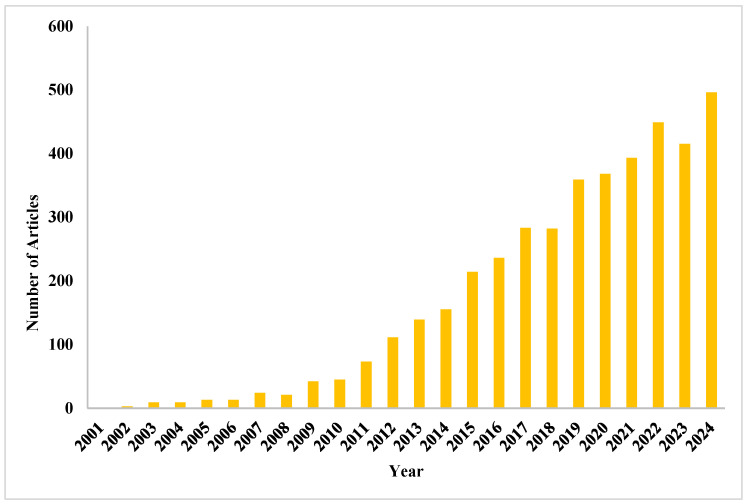
A growing interest in this multidisciplinary subject is demonstrated by the steady increase in the number of Scopus-indexed papers on nanomaterial-based sensors for detecting neuropharmaceuticals and neurotransmitters between 2001 and 2024.

Within the dynamic and continuously growing fields of neuroscience and pharmacology, there is a renewed focus on developing technologies to help us understand the complex biochemical nature of the brain and its function [1]. The difficulty of accurately identifying and tracking neurotransmitters and neurological pharmaceutical substances in real time has led to an evolution in favor of integrating nanotechnology. Utilizing the special qualities of materials at the nanoscale, nanomaterial-based sensing systems have become groundbreaking instruments with their unique sensitivity, selectivity, and versatility [2]. Figure 1 shows that, according to publication data taken from the Scopus database, research activity on nanomaterial-based sensors for neuropharmaceuticals and neurotransmitters has increased significantly and continuously over the past 20 years, demonstrating the field’s increasing scientific and clinical relevance.

Our understanding of neurochemistry is now much clearer, developing because of the incorporation of nanomaterials such as graphene, carbon nanotubes, quantum dots, nanoparticles, noble metal nanomaterials, hybrid polymers, etc. into optical and electrochemical sensing platforms [3]. These nanomaterial-based sensors facilitate the investigation of the neuroactive compounds that are found in pharmaceuticals, the human body, and the environment, giving scientists and medical professionals the instruments required to decipher the intricate molecular relationships within the brain to help treat, control, or monitor many disorders that affect the neurological system [4].

Because of their unique characteristics, such as huge surface area; unique electrical, chemical, and physical characteristics; and various easy, cheap synthesis methods, sensors based on nanomaterials provide a versatile method for neurochemical detection [5]. For example, carbon nanotubes have excellent electrical conductivity, which makes them perfect for real-time monitoring applications. In contrast, quantum dots have special optical properties that allow highly specific and sensitive detection of neurotransmitters. The two-dimensional structure of graphene provides an exceptional surface area for molecular interactions, which improves the accuracy of neuropharmaceutical compound detection even more [6].

This review aims to explore the complex field of sensing systems based on nanomaterials specifically designed to detect neurotransmitters and neuropharmaceutical compounds. We aim to shed light on these sensing technologies’ revolutionary effects on neuropharmacological research by clarifying the fundamental ideas driving them and investigating how they are applied in this domain. This review first discusses sensor fundamentals and then examines different nanomaterial classes, followed by examples of applications and future perspectives.

## 2. Sensor Systems in Neuroscience

A sensor is a biological or chemical structure device used to detect and identify a specific event and then translate it into a measurable signal. Sensors can be used to detect specific ions, molecules, or even changes in temperature, pH, etc. [7].

In neuroscience, it became necessary to develop sensors that can detect specific ions, neuromodulators, neurotransmitters, and some pharmaceuticals, as many neurological disorders and processes are related to them [8,9]. Unfortunately, the detection of such neurological agents is complex and cannot be performed precisely while using conventional methods such as spectrophotometry, liquid or gas chromatography, etc. This can be attributed to the numerous analytes that must be detected simultaneously inside extremely complex sample matrices and at high levels of spatial and temporal resolution [10].

There are different types of sensors, depending on the detection mechanism. Optical sensors, for example, mostly use fluorescent markers that bond specifically to a target molecule, resulting in fluorescence upon bonding [11]. Another sensing concept is electrochemical sensing, which is based on redox reactions and is currently more used and reliable than optical techniques [12]. Table 1 provides a comparison of optical and electrochemical sensing systems, along with some examples.

However, recent developments in nanotechnology have given neuroscientists access to invaluable new tools and materials. These include nanosensors made of metallic nanostructures, engineered fluorescent proteins, and organic molecules [7]. The general concepts of nanotechnology were defined by Richard Feynman in 1958 as considering the technology behind the process of creating machine tools using even smaller machine tools [24]. Because of the many characteristics that nanomaterials have over micro- and macroscale materials, such as high surface area, specific delivery, easy surface modification, flexibility, different synthesis methods, and unique chemical, electrical, optical, and mechanical properties, they have become favorable in many fields, especially the medical field [25,26]. These characteristics have created a wide array of applications for nanomaterials in medicine; nanomaterials are used in drug delivery systems, drug screening, therapeutic scaffolds, diagnostics, and developing sensors [3,27]. Nevertheless, among the first uses of nanotechnology is the development of various biological and chemical sensors [20].

### 2.1. Nanomaterial-Based Electrochemical Sensors

As was mentioned before, the unique chemical and biological characteristics of nanomaterials have become widely used in developing nanosensors. The use of nanobiosensors in the identification of precise anatomical locations or specific cell types in the human body is potentially growing in the fields of medical diagnostics and analysis. Table 2 summarizes the types of nanomaterials used for developing electrochemical sensors. The high sensitivity and ease of nanosensor miniaturization and functionalization may help create a new paradigm for clinical analytical instruments [1]. Numerous nanomaterial-based electrochemical sensing techniques have been developed for various biological and biomedical applications. Nanomaterial-based sensors used in the detection of neurological drugs are summarized in Table 3.

#### 2.1.1. Noble Metal Nanomaterials

The unique catalytic properties of metal nanomaterials result from their sizes, shapes, and synergistic effects. There are various types of metal nanomaterials, such as silver, gold, palladium, platinum, and bi- or trimetallic nanoparticles. Compared with carbon-based nanomaterials, metal nanoparticles are more selective because of the unique interactions between the metal and the target molecule; metal nanoparticles can alter and adapt their morphology or composition [1,5].

In a study performed by Chauhan et al. [91] acetylcholine, an important neurotransmitter for both the central and peripheral nervous system that plays a crucial part in movement, memory, and emotions, was detected using an acetylcholine biosensor. The sensor was prepared by coimmobilizing both acetylcholinesterase and choline oxidase on platinum nanoparticles in addition to a metallic organic framework-modified gold electrode. The platinum-nanoparticle-based sensor exhibited a low detection point of acetylcholine chloride of 0.01 μM with almost no interferences and a broad linear detection range of 0.01–500 μM. In another study by Sanghavi et al. [37] a copper (Cu(II)) complex along with silver nanoparticles as a modifier was used to prepare a biomimetic sensor for the determination of some catecholamines (e.g., dopamine, epinephrine, levodopa, etc.). For dopamine, a neurotransmitter that plays a crucial role in the function of cardiovascular and hormonal systems, the prepared sensor had a lowest detection limit of 35 nM. A more recent example is the AuNP-based electrochemical biosensor for glucose detection created by Zhang et al. [38], which achieved much better stability and sensitivity by immobilizing enzymes on a nanocomposite matrix. In clinical diagnostics, this illustrates how AuNPs continue to be crucial for improving electrochemical transmission.

#### 2.1.2. Metal Oxide Nanomaterials

Metal oxide nanoparticles are widely used in many different domains, including catalysis, electrochemistry, soft magnetism, sensors, and more. Metal oxide nanomaterials have the potential to considerably improve the sensitivity and/or selectivity of electrochemical analysis. They also have a high organic capture affinity, are relatively cheap, and have high electrocatalytic activity, which is why metal oxide nanoparticles have been used extensively for sensing a variety of analytes [92,93]. For example, Fan et al. [47] developed a titanium dioxide (TiO_2_) nanoparticle-based sensor for the detection of paracetamol (an analgesic drug). The sensor consisted of TiO_2_, graphene, and Nafion. TiO_2_ nanoparticles improved the electrochemical activity and response to paracetamol via the modification they caused to the chemistry of the surface of graphene sheets. The limit of detection of paracetamol using this technique was 0.21 μM. Mazloum-Ardakani et al. [94] developed an electrode of carbon paste modified with zirconium dioxide (ZrO_2_) nanoparticles. This electrode was prepared to detect and study norepinephrine, folic acid, acetaminophen, and their mixtures using electrochemical methods. The ZrO_2_-nanoparticle-modified electrode solved the problem of overlapping between the signals of the chemicals. The detection limits of norepinephrine, folic acid, and acetaminophen were 8.95 × 10^−8^, 9.86 × 10^−6^ and 9.12 × 10^−7^ M, respectively. Many more metal oxide nanoparticles have been used in electroanalysis, such as cerium oxide (CeO_2_), iron oxide (Fe_2_O_3_), copper oxide (CuO), nickel oxide (NiO) zinc oxide (ZnO), cadmium oxide (CdO), etc.

#### 2.1.3. Carbon-Based Nanomaterials

A key component of the sustainable development of nanotechnologies is the ongoing advancement of the materials used to create tools, technologies, and scaffolds for use in different fields. In this regard, special attention needs to be paid to carbon-based nanostructures, which are composed of high-purity carbon with different atomic hybridization or geometrical patterns [95]. These materials offer higher detection sensitivity than others due to their chemical stability, electrical conductivity, and improved mechanical strength [96]. One example of carbon-based nanomaterials is carbon nanotubes. The cylindrical tubular morphology of these tubes has allowed them to be easily functionalized and modified, facilitating their use in different medical and pharmaceutical applications. These tubes can be subdivided into single-walled nanotubes (SWCNTs) and multiwalled nanotubes (MWCNTs). While the first consists of cylindrical tubes 0.4–2.5 nm in diameter formed from graphene sheets, the second has many layers of stacked graphene sheets formed into cylinders with versatile diameters that can reach 100 nm [97]. Both SWCNTs and MWCNTs exhibit high conductivity, adjustability, and surface-area-to-volume ratios. However, it should be noted that there are differences that should be considered when producing for a specific application, as SWCNTs’ production is more expensive, needs catalysts, and is more flexible than that of MWCNTs [98].

Other examples of carbon-based nanomaterials are graphene and graphene oxide, nanodiamonds, buckypaper, fullerenes, etc. Carbon-based nanomaterials are used in different fields. For instance, a glassy carbon electrode coated by an MWCNT was developed by Wu et al. for the detection of procaine, which is a local anesthetic. After some optimization steps, the detection limit was found to be 2 × 10^−7^ mol/L [99]. Mokhtari et al. prepared a carbon paste electrode and vinylferrocene/MWCNTs to detect morphine. Morphine is an opiate analgesic that is used for patients with severe pain but can cause damage to the central nervous system. The detection limits of 0.09 μmol L^−1^ could be achieved for morphine. The prepared sensor was successfully applied for morphine detection [38].

#### 2.1.4. Polymeric Nanomaterials

In biomedical applications, polymeric nanoparticles are useful, especially in drug delivery and biosensing. These consist of structures such as polymer nanogels, polymer micelles, and polymer dots (Pdots). Their precise design and optimization are made possible by their changeable physical and chemical features. A thorough examination of the polymers employed, especially for the fabrication of nanosensors is provided in Section 3.

Sensory polymers are polymer families that can bond with a target molecule via a selective interaction. Some of these\polymer families are conductive polymers, molecularly imprinted polymers, hybrid polymers, dendrimers, etc. [100]. The main advantage of polymeric nanomaterials is that they can be adjusted in several ways, as they have surface modification potential that can be functionalized according to the target biomolecule. They provide excellent kinetic control [101]. Because of their sensitivity, selectivity, and linearity, polymeric nanomaterials have been used to detect different biomolecules (proteins, metabolites, DNA, etc.).

Dendrimers are synthetic, polymer-based molecules made of monomers with three or twice as many peripheral groups in each layer of branching units as in the original monomer (they resemble foam balls). Targeted gene and medication delivery has great potential due to a dendrimer’s void area, ease of manufacturing and modification, and size control. Enhancements are required in the areas of cytotoxicity profiles, biocompatibility, and biodistribution [102]. Dendrimers contain a high ratio of amine groups, allowing them to detect highly sensitive biological analytes. Zhang et al. [103] used a sensor that was developed by Miodek et al. [104] which consisted of polypyrrole-coated MWCNTs and poly-(amidoamine) dendrimers, for the detection of paracetamol. The sensor exhibited a great catalytic activity towards paracetamol, as the limit of detection appeared to be 1.0 × 10^−7^ M, with a broad detection range between 3.0 × 10^−7^ and 2.0 × 10^−4^ M.

Conducting polymers have been used in chemical, biological, and gas sensing, along with other sensing techniques, because of their unique electronic properties. Unnikrishan et al. [70] fabricated a sensor that consisted of a glassy carbon electrode modified by multiwalled carbon nanotube–polyethyleneimine to detect chlorpromazine, which is a drug used to manage psychotic conditions by controlling agitation, excitement, and other symptoms. Polyethyleneimine facilitated the disperse of carbon nanotubes as well as serving a prominent role in increasing the intensity of the detection signal because of to its high conductivity. In this study, when compared with bare glassy carbon, the prepared electrode showed a 15.7-fold increased signal peak. The limit of detection by the fabricated electrode appeared to be 10 nM.

Molecularly imprinted polymer (MIPs) are prepared by the polymerization of a monomer and a template target molecule. Later, the template molecule is separated to create a polymer with a receptor site specific to the target molecule [105,106]. The extreme selectivity of MIPs and the fact that a large number of monomers can be employed to identify certain target molecules, providing a wide range of sensory options, are what make MIPs such valuable sensors [107]. MIPs as sensors have been used to detect different types of molecules, such as steroids, amino acids, metals, ions, peptides, and many more. Prasad et al. [108] made an epinephrine-imprinted polymer-based electrochemical sensor. The procedure of producing the sensor included dispersing epinephrine into multiwalled carbon nanotubes bearing terminal monomeric units. The polymerizing process was carried out at 60 °C. After polymerization, the resulting MIP was completely freed from the template molecules, creating a binding site. This MIP sensor was made to be selective for epinephrine. The LOD was found to be 0.1 nM. Nanomaterial-based optical and electrochemical sensors have been developed to detect neurotransmitters because of their efficient electrochemistry and biological significance. Table 4 describes neurotransmitters and their nanomaterial-based detection methods in the literature.

**Figure 2:**
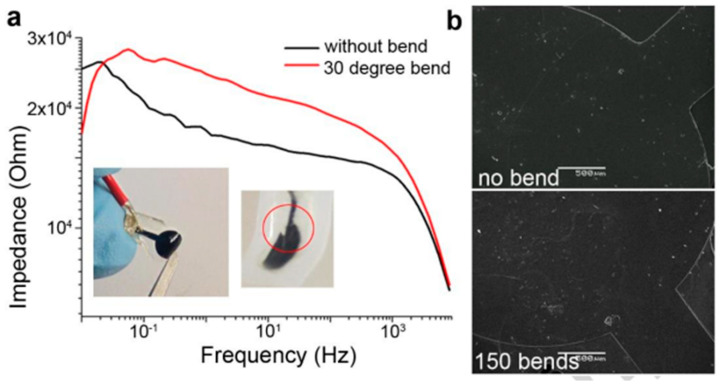
(**a**) Mechanical stability under stress; (**b**) SEM of the surface of PEDOT:PSS [128].

Poly (3,4-ethylenedioxythiophene):poly(styrene sulfonate) (PEDOT:PSS) sensors on a biodegradable and flexible silk protein fibroin support were developed by Pal et al. The fabricated biosensors exhibited excellent and sensitive nonspecific detection of ascorbic acid and dopamine. The effect of mechanical stress on the sensor was studied to prove the feasibility of the sensor in flexible systems. After being bent to about 30°, the conductive patterns were identified using electrochemical impedance spectroscopy. The material’s impedance increased slightly because of the bend, but the order stayed the same (Figure 2a). Separately, bending (30°) and releasing were applied repeatedly to conductive patterns. The charge storage capacity values only slightly decreased to 150 cycles as the stress cycles increased. SEM pictures of the surfaces revealed no delamination or damage to the composite (Figure 2b), demonstrating high-stress stability and the capacity to maintain electrical and electrochemical characteristics under challenging mechanical circumstances [128].

**Figure 3:**
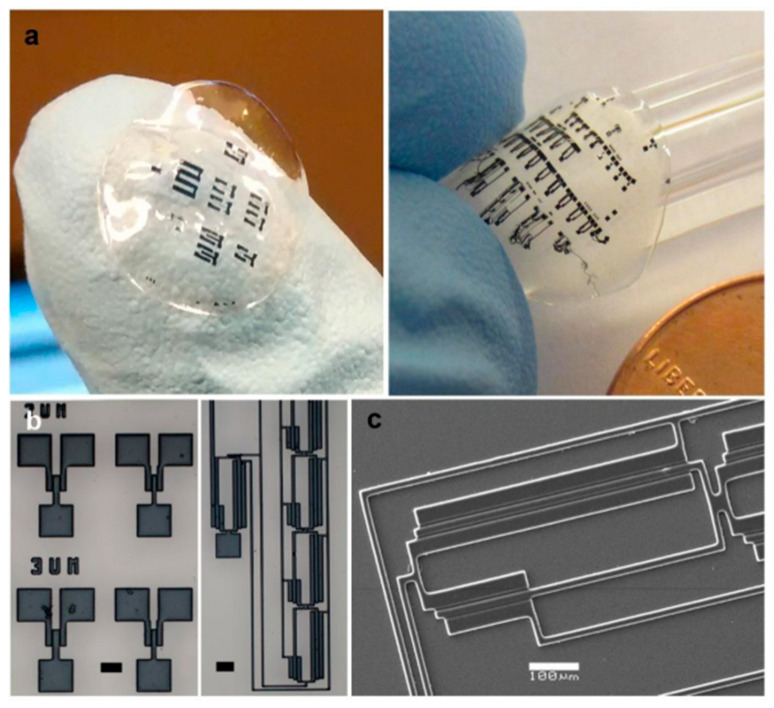
Formation of complex microstructures using photolithography: (**a**) large-area micropatterns of PEDOT:PSS formed on silk fibroin sheets; (**b**) optical micrograph images of PEDOT:PSS micropatterns on glass; (**c**) SEM images of PEDOT:PSS micropatterns on glass [128].

Then, a photoreactive conductive sensing ink was formed by combining PEDOT:PSS with ericin protein photoresist (SPP) and casted on fibroin protein photoresist (Figure 3a), where a film the size of a contact lens is used to demonstrate microscale patterning. High-fidelity structures were generated by this process (Figure 3b). SEM imaging confirmed relatively smooth and extremely clear patterns with feature sizes as small as ~5 µm (Figure 3c) [128].

#### 2.1.5. Bionanomaterials

The development of nanotechnology along with biotechnology has led to the development of nanomaterial-based electrochemical biosensors that focus on the detection of proteins/enzymes, peptides, biopolymers, and nucleic acids. Because of their biocompatibility and effective sensing performances in terms of high selectivity and sensitivity, bionanomaterial-based electrochemical biosensor systems have been successfully produced and used in different fields. The synthesis, functionalization, and integration of bionanomaterials have advanced to the point where biological systems can now readily attain the required level of selectivity, sensitivity and rapid reaction, and recovery. Aptamers and DNA nano structures are excellent examples of bionanomaterials used for developing sensors [1].

Aptamers are nucleic-acid-based molecules that consist of single strand that can be functionalized to have a selectivity to specific molecules. Farjami et al. [129] developed a sensor based on RNA aptamer that was immobilized on an Au electrode modified with cysteamine. The sensor was specifically developed to detect dopamine in the presence of other neurotransmitters, which is why an aptamer-based sensor was developed and used. The sensor allowed the detection of dopamine in a wide range between 100 nM and 5 μM without any interference from other neurotransmitters such as epinephrine, tyramine, or many others. The response time of the sensor was less than a second, and its selectivity reached 62 nA μM^−1^ cm^−2^. Similarly to aptamers, DNA nanostructures have been used to develop biosensors because of their low cost, simple synthesis, and high selectivity. Jankowska-Śliwińska et al. [130] developed a DNA-modified gold paste electrode. The sensor was used for the detection of imipramine, which is a tricyclic antidepressant. The DNA-modified sensor exhibited better selectivity and limit of detection than a bare gold paste electrode.

**Figure 4:**
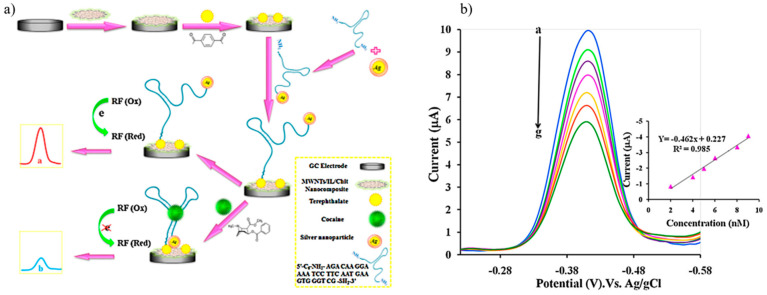
(**a**) The preparation and mechanism of response of the electrochemical aptasensor for the detection of cocaine; (**b**) the differential pulse voltametric responses of the novel aptasensor after being incubated for 45 min with different concentrations of cocaine [131].

Roushani and Shahdost constructed an aptasensor by covalent immobilization of aptamer-functionalized Ag nanoparticles on a MWCNT/ionic liquid/chitosan nanocomposite. Riboflavin (RF) was used as the redox probe in the electrochemical aptasensor for the purpose of diagnosing cocaine. As described in Figure 4a, the aptasensor was created by first coating a glassy carbon electrode with MWCNTs/ionic liquid/chitosan nanocomposites to increase the active surface area of the electrode. Second, the aptamer molecules were immobilized by terephthalate. Third, the amide coupling of aptamer molecules to the amine groups of chitosan in the nanocomposite on the electrochemical surface was achieved by adding a 5-amino3-Ag-nanoparticle-functionalized aptamer. When cocaine did not exist, the sensor exhibited a well-defined signal due to the reduction of RF. When cocaine was introduced, its selective binding to the Ag nanoparticles caused a steric hindrance, which caused a limitation between the aptamer and RF redox probe and thus a decrease in the current peak. In the presence of varying cocaine concentrations, well-defined differential pulse voltametric responses were exhibited, and the peak current signals associated with RF decreased as cocaine concentration rose (Figure 4b) [131].

#### 2.1.6. Two-Dimensional Nanomaterials for Neurosensing

Graphene, MoS_2_, black phosphorus, and MXenes are examples of two-dimensional (2D) nanomaterials that have shown promise in the development of sensitive and selective biosensors. For the electrochemical and optical detection of neurotransmitters and neuropharmaceutical substances, their enormous surface area, adjustable electronic characteristics, and ease of surface functionalization make them perfect. To detect dopamine and serotonin with submicromolar sensitivity, graphene oxide, for example, has been functionalized with gold nanoparticles and employed in voltametric sensors. Through synergistic conductivity and adsorption capabilities, MXene-based platforms—in particular, Ti_3_C_2_T_x_—have been used to detect antiepileptic medications such as carbamazepine. These results have been corroborated by recent studies that emphasize the biocompatibility and adaptability of 2D materials when combined with flexible substrates and microelectrode arrays [132,133].

### 2.2. Nanomaterial-Based Optical Sensors

Single-molecule recognition is currently a well-established capability of optical technologies. The biological recognition materials or probes bind to the fluorescent markers, producing a fluorescence signature from the probe-target interaction [11]. Nanomaterial-based sensors proved to have enhanced optoelectronic properties that have provided great benefits to the biosensing field. One type of nanomaterial used in this context is fluorescent nanomaterials, which have proven to have superior optical properties to those of conventional fluorophores, allowing them to be used to develop nanosensors. In this context, SWCNTs became very famous because they have a bandgap and fluorescence in the near-infrared region. Each SWCNT’s NIR fluorescence is determined by its diameter and chiral vectors; hence, within the same surrounding medium, different chiral SWCNTs exhibit different sets of optical transitions. This phenomenon also depends on charge transfer [134]. Cha et al. [135] prepared a novel optical biosensor for real-time, noninvasive detection of insulin utilizing near-infrared fluorescent SWNTs functionalized with target-recognizing aptamer DNA. Insulin-binding aptamer was used for this purpose. As a molecular recognition element, the photoluminescence (PL) emission of aptamer-coated SWNTs was controlled upon selective binding to target molecules. This property was utilized to detect insulin. Nanotube PL was quenched upon insulin identification by a photoinduced charge transfer mechanism. The diffusion–reaction rate was found to be kr = 0.129 s^−1^, and the quenching rate was kq = 5.85 × 1014 M^−1^ s^−1^. Plasmonic gold nanoparticle (AuNP)-based optical biosensors have garnered attention recently because of their exceptional sensitivity and multiplexing capabilities. Jiang et al. [136] described a plasmonic sensor that uses AuNPs for ultrasensitive biomarker detection in complicated biological samples, with detection levels as low as subfemtomolar. In a similar vein, Patel et al. [137] employed DNA aptamer-functionalized AuNPs for the colorimetric detection of cancer biomarkers, achieving both high selectivity and visual detection capacity. The development of a multiplexed nanoplasmonic biosensor by Soler et al. [138] that incorporates AuNP-based microfluidics for the simultaneous, label-free detection of numerous neurological biomarkers in clinical samples shows great promise for point-of-care applications and advanced diagnostics. These studies show how AuNPs are becoming powerful optical probes for real-time, label-free diagnostics.

Quantum dots (QDs) are also an example of fluorescent sensors. QDs emit narrow emission spectra and broad absorption (from ultraviolet to infrared) in size-dependent ways. Because of their single excited and varied emission wavelengths, QDs attracted great interest in biosensing applications [139]. QDs consist of a semiconductor core and are often covered with a shell [140]. Freeman et al. [141] synthesized Cd-Se-ZnS quantum dots (QD) and functionalized them with a boronic acid ligand for the purpose of the detection of dopamine. Boronic acid can bind with the catechol part of the dopamine molecule. Dopamine was conjugated with an organic dye. The detection limit of dopamine using this method was found to be 1 × 10^−6^ M.

Another type of nanomaterial used in this context is engineered proteins. These are very promising sensors in the field of neuroscience. This technique is based on utilizing neurotransmitter receptors as highly specific sensors. A fluorescent sensor for glutamate was genetically encoded by Okumoto et al. [142] as one of the first sensors of its kind. It consisted of two fluorescent proteins and a bacterial glutamate-binding protein. Förster resonance energy transfer (FRET) is dependent on the glutamate binding protein’s conformation. Therefore, glutamate can be detected by alterations in the FRET signal. The highest-affinity sensor mutant yielded a Kd of 630 nM.

Other optical nanomaterial-based sensors include organic fluorophores. Their unique photophysical characteristics, small size, and cheapness made them valuable for the synthesis of biosensors. Secor and Glass [143] designed a sensor based on boronic acid and coumarin aldehyde for the detection of dopamine and other catecholamines. The mechanism of the detection involved the sensor bonding with catecholamines, forming an iminium ion along with the amine in addition to a boronate ester along with the catechol, followed by the internal hydrogen bond causing a colorimetric response with high selectivity.

**Figure 5:**
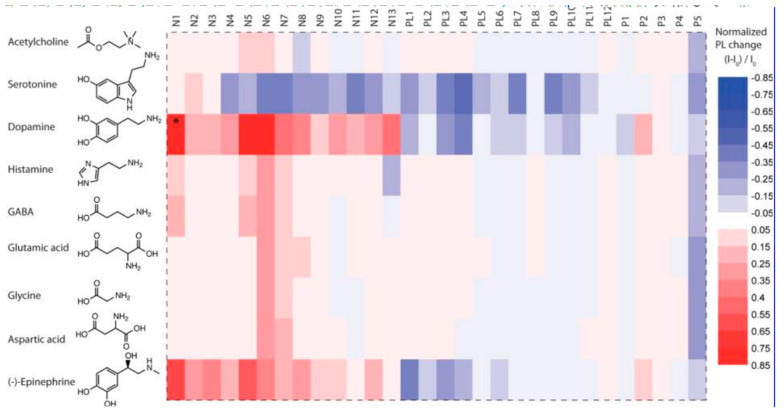
Fluorescence responses of polymer–SWCNT conjugates (x axis) to neurotransmitters (y axis) [144].

Information processing in neural networks relies heavily on changes in the concentration of neurotransmitters both spatially and temporally. Thus, neurotransmitter sensors are crucial instruments in the field of neuroscience. Corona phase molecular recognition was used by Kruss et al. [144] to identify adsorbed polymer phases on fluorescent SWCNTs, which enabled the selective detection of neurotransmitters. The optical responses of various SWCNT–polymer hybrids to different neurotransmitters are shown in Figure 5.

## 3. Polymers Used for the Synthesis of Nanosensors

Polymers that are most frequently used in the creation of nanosensors are categorized and described in this section. They can be either natural or synthetic, and they have a variety of uses, from providing structural support to improving the specificity and sensitivity of sensors. Chitosan, cellulose, and their derivatives are examples of natural polymers, whereas polyvinyl alcohol (PVA), polyethylene glycol (PEG), polypyrrole (PPy), and polyaniline (PANI) are examples of synthetic polymers. Moreover, polymeric nanostructures such as micelles and nanogels, which were discussed in Section 2.1.4, are used to encapsulate sensing agents, enabling better stability and targeted delivery within sensing systems.

In order to prepare different kinds of sensors for different kinds of molecules, especially for neuro-related molecules, the most necessary and important building blocks are polymers. In this section the most important sensory polymer families will be discussed. Sensory polymers are those that have the ability to respond to different stimuli, such as electromagnetic pulses, temperature change, bonding to molecules, etc. This response can be expressed in different ways, such as fluorescence, changes in color or shape, etc. [145]. These polymer families include MIPs, polymers with chiral motifs, hybrid polymers, conductive polymers, acrylic polymers, etc.

### 3.1. Molecularly Imprinted Polymers

This technology is based on imprinting template molecules in a polymeric organic/inorganic matrix using template–monomer complexes and covalent and noncovalent interactions. The molecular imprinting technique allows for the spatial organization of specific recognition sites. Following crosslinking copolymerization, the steric and chemical information of the imprinted molecules is left behind when templates are removed from the crosslinked matrix, creating recognition cavities corresponding to the size, shape, and functionality of templates. Therefore, by interacting specifically with these imprinted sites, the target species can rebind into the molecularly imprinted polymers (MIPs) in a selective manner [146]. Because of their high selectivity, stability, simplicity, affordability, and versatility in applications such as clinical diagnostics, food analysis, and drug monitoring, MIP-based sensors have become increasingly popular as specific sensing materials after Andersson et al. [147], originally proposed using optical surface ellipsometry to monitor the specific binding of vitamin K1 to a surface-imprinted silicon surface. MIPs are becoming more popular in different medical and industrial fields. The dual ability of MIPs to function as transduction and recognition elements, i.e., to specifically bind target analytes in their ability to act as transduction elements and produce output signals for detection, distinguishes MIP-based sensors from other types of sensors. MIPs can specifically bind these molecules, which can include ions, biomolecules, atoms, complexes, microorganisms, etc. [148]. The main disadvantage is that developing MIPs is quite slow because of the limited number of monomers that are used in molecular imprinting. There are also different ways to prepare MIPs, but the main two are covalent and noncovalent imprinting. Both techniques involve a template molecule, a functional monomer, an initiator for the polymerization, a cross-linker, and a solvent. In covalent imprinting, it is difficult to reach a thermodynamic equilibrium because of the strong covalent interactions formed, which cause slow binding and detachment. However, in noncovalent imprinting, the bonds that form are van der Waals forces, hydrogen bonding, and π–π interactions [149]. Functional monomers play an important role in providing suitable functional groups for binding with the template prior to polymerization. One of the most famous functional monomers is methacrylic acid because it has both hydrogen-donating and -accepting properties. Other famous functional monomers include acrylic acid, 4-vinyl benzaldehyde, acrylamide 2-hydroxyethyl methacrylate, and many more [150]. Wang et al., [151] developed an electrochemical multiplex immunoassay that used dual-template magnetic MIPs as capture probes and recombinant apoferritin-encoded metallic nanoparticles as labels to detect alpha fetoprotein and the carcinoembryonic antigen simultaneously. In order to prepare the labels, recombinant apoferritin was loaded, and primary antibodies were immobilized separately using in situ growth of gold nanoparticles on graphene. These findings implied that the multiplexed immunoassay that was suggested might be useful for clinical screening of additional biomarkers.

### 3.2. Hybrid Polymers and Polymeric Nanocomposites

Both hybrid polymers and polymeric nanocomposites have two-phased structures that consist of organic and inorganic phases. In hybrid polymers, these phases are linked together with covalent bonds to create a one phase component. However, the preparation of polymeric nanocomposites requires a polymeric mixture and inorganic particles with diameters between 1 and 100 nm [152]. The inorganic component increases the complexity and functionality of the structure, which is a result of being incorporated as a single component in a multilevel structured material and synergistically interacting with the organic component. Polymer nanocomposites exhibit better physical and chemical properties than either pure polymer matrices or composites containing larger-sized components. The relative crystallinity or amorphous structure of the polymer matrix and the interaction between the filler and the matrix are two of the many factors that affect the effects of the nanoparticles [153].

**Figure 6:**
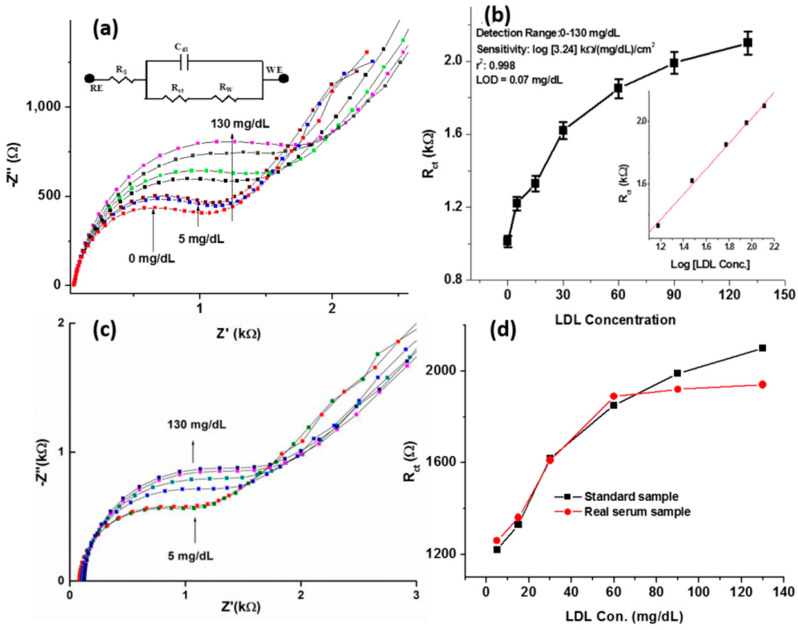
Response analysis of the sensor at 0.1 V in relation to LDL concentration: (**a**) plot of the (initial response) R_ct_ value vs. LDL concentration; (**b**) sensor calibration logarithm plot; (**c**) plot of the response of the sensor to LDL levels of 5–130 mg/dL in blood serum samples; (**d**) plot comparing the R_ct_ values and LDL concentrations for both standard and real samples [154].

**Figure 7:**
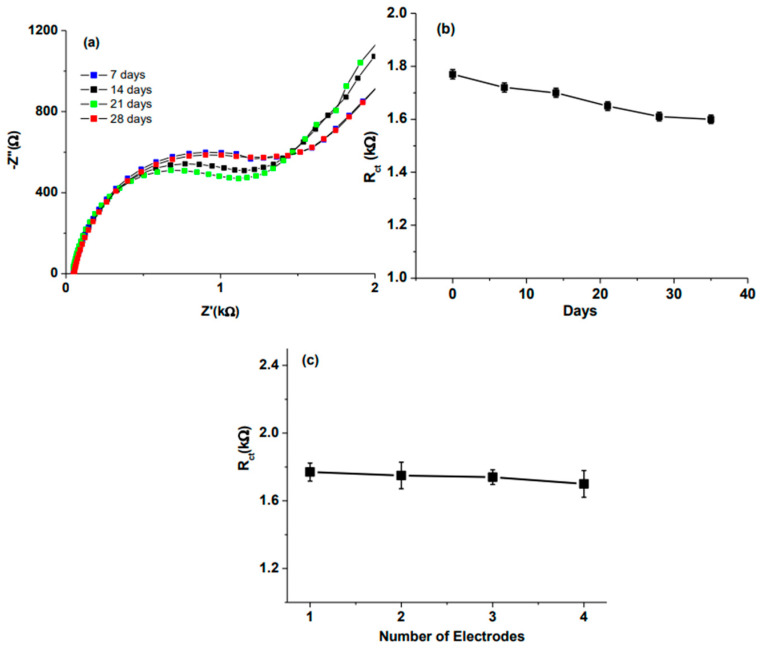
The fabricated sensor was evaluated for 35 days, and the following data were obtained: (**a**) electrochemical impedance spectra with the presence of LDL molecules (60 mg/dL); (**b**) the relationship between the number of days and the obtained R_ct_ values; (**c**) sensor responses with different numbers of repeated electrodes [154].

Ali et al. [154] developed an anti-apolipoprotein B 100 (AAB) functionalized mesoporous few-layer reduced graphene oxide and nickel oxide (rGO-NiO) nanocomposite sensor for the detection of low-density lipoprotein (LDL) molecules. The sensitivity of the sensor was found to be 510 Ω (mg/dL)^−1^ cm^−2^, with a detection range of 0–130 mg/dL. This sensor could be used to detect a low concentration of LDL, with an LOD of 0.07 mg/dL, compared with other reported biosensors that are used for the same purpose (Figure 6). After 35 days of analysis, the response of the fabricated sensor decreased to 90.3% of the initial response (R_ct_). A low relative standard deviation of the means indicated that the sensor exhibited excellent stability. This may be attributed to the strong covalent bonds of AAB on the functional mesoporous rGO-NiO composite. The experiment was repeated with four different sensors, which were prepared under the same conditions. The standard deviation of these electrodes appeared to be 4%, which confirmed the reproducibility of these sensors (Figure 7).

### 3.3. Acrylic Polymers

Conventional polymeric materials, including acrylic polymers, have been studied for many years. Because of their adaptability, these polymers can be used as sensory materials. In addition, a wide range of sensory units can be chemically attached to their structure, which offers them several benefits. The most common types of acrylic polymers used in sensory applications are acrylamide derivatives and esters of acrylic or methacrylic acid and their copolymers [155].

### 3.4. Conductive Polymers

Main polymer chains with alternating simple and multiple bonds forms conductive polymers. These polymers are semiconductors or electrical insulators in their natural state, but doping them makes them highly conductive materials. They also exhibit luminescence, a physical characteristic that can be utilized as an output signal in sensory devices along with the electrical signal produced by semiconductivity. Polypyrrole, polyaniline, and derivatives of fluorene are some examples of conducting polymers [156].

**Figure 8:**
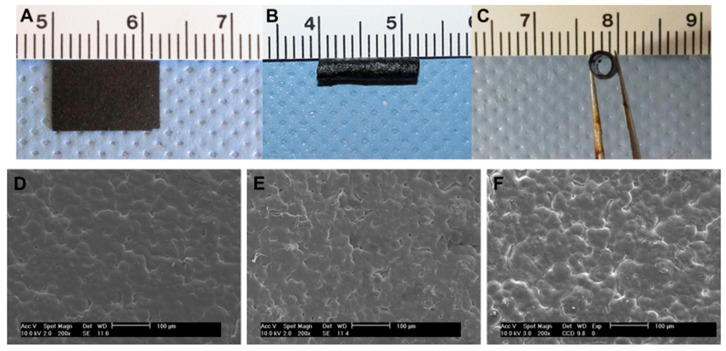
(**A**) the PPY/PDLLA film; (**B**) and (**C**) the PPY/PDLLA conduit; (**D**–**F**) the surface morphology of the 5%, 10%, and 15% PPY/PDLLA conduits, respectively [157].

Polypyrrole (PPY)/poly(D, L-lactic acid) (PDLLA) conduits were fabricated by Xu et al. [157] in specific sizes and with different amounts of PPY. Actual and SEM images of the PPY/PDLLA films and conduits are shown in Figure 8.

**Figure 9:**
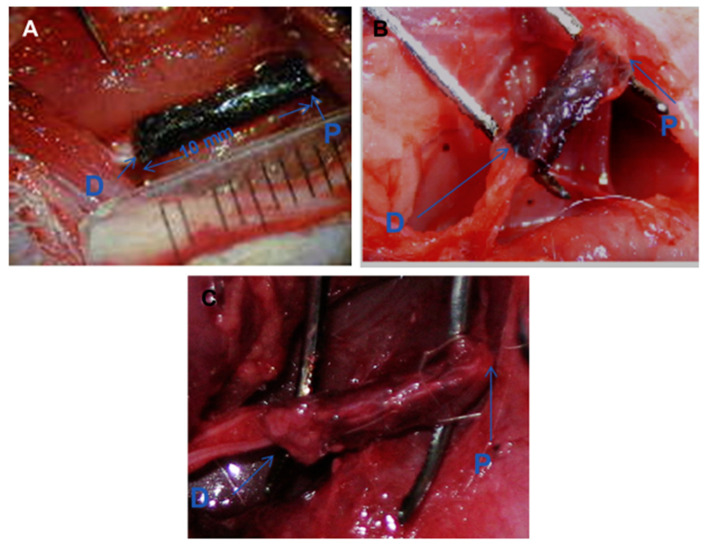
Intraoperative photographs of the PPY/PDLLA nerve conduits (**A**) immediately after implantation, (**B**) 3 months postsurgery, and (**C**) 6 months postsurgery [157]. (“P” signifies the proximal end and “D” signifies the distal end).

Figure 9 shows the degradation of the PPY/PDLLA conduit at different times after implantation. Significant levels of degradation were observed over time. The conduit became thin and then crisp after 3 months, albeit maintaining the integrity of the lumen and wall. Even greater degradation was recorded after 6 months, but significant regeneration had already occurred by that time, which indicates that the conduit successfully met the application it was used for [157].

**Figure 10:**
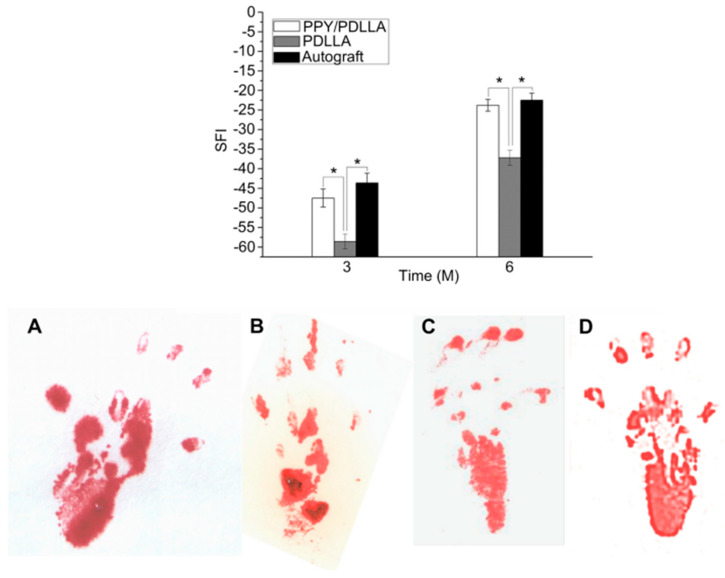
Recovery of sciatic nerve function. Sciatic function index (SFI) as a function of implantation time (**top**). Footprint stamps in walking track analysis after 6 months of implantation (**bottom**): (**A**) PPY/PDLLA; (**B**) PDLLA; (**C**) autograft; (**D**) normal left leg [157]. (*: showing statistically significant differences between two groups).

Sciatic function index (SFI) is a measure of the sciatic nerve function. A value of SFI that is close to 0 is an indicator of normal function, while a value close to 100 implies a total impairment. As shown in the top part of Figure 10, after 3 and 6 months of implantation, there was a significant difference between the PPY/PDLLA group and the PDLLA group, while there was no significant difference between the PPY/PDLLA group and the autograft group (epineurial coaptation). A walking track analysis of all nerve conduits six months after implantation is depicted in the bottom part of Figure 10. The PPY/PDLLA group’s footprints were wider and shorter than the PDLLA group’s, and they also demonstrated a higher degree of improvement in toe spreading. Remarkably, the autograft group’s outcomes were comparable to those of the PPY/PDLLA group [157].

### 3.5. Polymers with Chiral Motifs

In the last few years, chiral ligands have been incorporated into polymer main chains to attain a specific chiral recognition of some chiral analytes. Chirality adds an optical response property to the polymer. This can be used in making polymeric sensors that are based on the alteration of optical properties due to the detection of the target species [23]. However, studies have shown that using one type of these kind of polymers may not always achieve the wanted results in terms of specificity, high resolution, obtaining a good signal, and reproducibility. Therefore, it is recommended to use more than one polymeric material simultaneously.

## 4. Application of Sensor Systems in Neuroscience and Neurotransmitters

### 4.1. Sensors in Neuroscience

The quickly developing field of nanotechnology offers accessible and useful tools for studying the nervous system in both its healthy and diseased states [7]. These tools include probes and sensors that are based on nanostructures. Some of these nanostructures are quantum dots, carbon-based structures nanoparticles, nanoprobes, magnetic nanoparticles, plasmonic nanoparticles, and hybrid nanotools [158]. Since nanomaterials allow for functional flexibility, self-assembly, and the emergence of novel electrical, optical, and catalytic properties at this scale, they are an important driver behind the widespread use of electrochemical methods in detection systems. Therefore, adapting nanomaterials in sensor systems has increased sensitivity, selectivity, and versatility [20]. In neuroscience, nanomaterial-based sensory systems are currently being developed and used for the detection of (1) ions and ion channels, which are key modulators of numerous neuronal processes; (2) neural activity; (3) neurological drugs; and (4) neurotransmitters and neuromodulators. Detecting and monitoring these neuroagents and the dynamics in the brain is an efficient strategy to learn and interpret neural activity, which facilitates the understanding of neurological disorders, such as Alzheimer’s disease, epilepsy, Down syndrome, and Parkinson’s disease [156].

### 4.2. Sensors for Neurological Drugs

Depending on the target drug’s structure, particular sensor interfaces are frequently required. New sensors are always being developed to increase stability and sensitivity and to reduce the detection limits of the analysis. Sensor systems for several important pharmaceutical compounds, such as anesthetics, analgesics, antiepileptics, anti-Parkinson’s, psycholeptics, and other nervous system drugs, have recently advanced [5,20].

### 4.3. Sensors for Neurotransmitters

Neurotransmitters are chemicals that serve as messengers to exchange information by cells in synaptic transmission. They play a major role in regulating brain functions. The detection of neurotransmitters is necessary to understand the mechanisms of the brain and to cure and treat diseases [126]. Epinephrine, norepinephrine, dopamine, glutamate, serotonin, acetylcholine, GABA, and glycine are some of the most famous neurotransmitters [94]. Neurotransmitters play an important role in regulating many processes in the human body, such as sleep, temperament, appetite, feelings, learning, attentiveness, memory, and several other cognitive activities [159]. Furthermore, some studies have suggested that unusually high levels of neurotransmitters are related to substance use. Lack of or increases in these neurotransmitters can cause different disorders; for example, Parkinson’s syndrome is connected the lack of dopamine, while schizophrenia is influenced by its accumulation [160].

## 5. Discussion

Nanomaterials have been used widely in different medical fields, such as drug delivery, biosensors, tissue engineering, etc. Here, we discussed the use of nanomaterial-based sensors in the detection of neurological molecules and drugs that affect the functions and health of the neurological system. Their small sizes, unique chemical and physical properties, huge surface area, and diversity facilitate the real-time precision that made them so popular in this field. These nanomaterial-based sensors also help with understanding and treating different neurological disorders such as Parkinson’s and Alzheimer’s. Despite these advances, there are still several limitations and challenges that need to be addressed. Biocompatibility is a major concern, since certain nanomaterials might trigger immunological reactions, inflammation, or long-term toxicity, which would make in vivo usage unsafe. Furthermore, signal drift over time, which can be brought on by surface fouling, material degradation, or environmental variations, might jeopardize the stability and reliability of sensor data, particularly in continuous or long-term monitoring. Interference with complex biological matrices, such as blood, cerebrospinal fluid, or tissue samples, is another major barrier. Nonspecific interactions and background noise might lower the sensors’ sensitivity and selectivity. Furthermore, in order to guarantee accuracy, reproducibility, and regulatory compliance, the transfer of these sensors from laboratory research to clinical practice necessitates strong calibration and validation procedures. The incorporation of nanomaterial-based sensors into standard clinical workflows will continue to be restricted unless these interrelated technical and biological issues are resolved [161,162,163].

Future directions for nanomaterial-based sensors in neuroscience include multimodal strategies, blending various nanomaterials, and fusing sensors with already-tried imaging methods. The fusion of different methods has proven to enhance the sensitivity and specificity of neurological assessments. In summary, nanomaterial-based sensors represent a promising area of neuroscience that has the potential to revolutionize our knowledge of and approaches to treating neurological illnesses. It is possible that further developments, moral issues, and the incorporation of these sensors into neuroscientific procedures will transform how we investigate and treat intricate neurological disorders. The integration of nanomaterials into biosensors has significantly improved our ability to detect neuroactive compounds and pharmaceutical agents with exceptional sensitivity. One of the most promising technological developments in this field is digital biomarker detection. Recent studies have shown that digital sensing platforms such as IRIS, when combined with nanoparticle-assisted strategies, offer a powerful method for quantifying biomolecules at ultra-low concentrations with single-molecule precision. Specifically, the use of gold nanoparticle tags has been demonstrated to enhance optical signals and reduce steric hindrance, enabling the direct visualization and counting of individual binding events. This has led to subfemtomolar detection sensitivity across wide dynamic ranges in both serum and whole blood, supporting the clinical utility of digital assays in allergy diagnostics, infectious disease detection, and cancer biomarker analysis [164,165,166,167]. We think that hybrid nanostructures that combine several functions will probably be the driving force behind the next generation of biosensors. Gold–silver core–shell nanoparticles, for example, have complementary optical and catalytic characteristics that make them ideal for dual-mode sensing platforms [168]. Because of their high surface area and electron mobility, graphene–AuNP hybrids and MXene-based composites are becoming attractive options for high signal-to-noise detection, especially in complex fluids [169,170]. Furthermore, a new class of wearable, intelligent, real-time biosensors for ongoing health monitoring may be introduced by combining these nanomaterials with microfluidic devices and AI-based data analysis.

## Figures and Tables

**Table 1:** Comparison between optical and electrochemical sensing systems.

Sensor Types	Advantages and Applications	Disadvantages	Examples	Refs.
Electrochemical	-Simple sample preparation and fast sampling time -High selectivity resulting from electrical signals at distinctive formal potentials -Can analyze various biological matrices -Can provide significant information about the way a drug is metabolized at specific dosage levels and its interactions with living cells	-Limited selectivity in complex biological matrices -Signal drift and electrode fouling -Stability issues in vivo applications -Challenges in calibration and reproducibility	-Carbon-based electrodes -Chemically modified electrodes	[13,14,15,16,17,18]
Optical	-Devices can be miniaturized -Lacks direct contact (non-invasive) -High spatial resolution -Nondestructive	-Photobleaching and signal instability -Interference from autofluorescence -Limited penetration depth	-Fluorescent carbon nanotubes -Quantum dots -Engineered proteins -Enzyme-conjugated nanoparticles -Small organic fluorophores	[6,19,20,21,22,23]

**Table 2:** Types of nanomaterials used for developing electrochemical sensors.

Nanomaterial	Example	Functional and Structural Features	Refs.
Noble metal nanomaterials	-Au nanoparticles	-High chemical stability -Easy preparation and fabrication methods -Wide electrochemical potential range -Biocompatibility -High catalytic activity	[28,29,30,31,32,33]
	-Ag nanoparticles	-High conductivity -Biocompatibility -Amplified electrochemical signal	
	-Platinum nanoparticles	-Distinctive electrocatalytic and electronic properties	
	-Palladium nanoparticles	-High catalytic and sensor activity	
Metal oxide nanomaterials	-Cerium oxide nanoparticles	-Enzymes and proteins can be easily immobilized on its surface -High catalytic activity	[34,35]
	-Copper oxide nanoparticles	-Various valence states -Tunable electron-transport performance -High surface area	
	-Magnetic nanoparticles	-Highly active and accessible surface are -Unique electron-transfer behavior	
Carbon-based nanomaterials	-Carbon nanotubes	-Their chemical properties can be easily modified and conjugated due to their tubular structure	[1,36,37,38]
	-Graphene	-High sensitivity -High selectivity -Low over potential -High electrocatalytic activity -Wide potential range	
Polymeric nanomaterials	-Dendrimers	-Structurally consistent and organized -Accurate -Biocompatible	[1]
	-Conducting polymers	-Unique electronic properties	
	-Molecularly imprinted polymers	-High selectivity	
Bionanomaterials	-Aptamers	-High affinity -High selectivity	[1]
	-DNA nanostructures	-Reusability -Simple synthesis process -High affinity and selectivity -Low cost	

**Table 3:** Nanomaterial-based sensors used in the detection of neurological drugs.

Drug Family	Drug/Compound Name	Some Nanomaterial-Based Sensor System Used for Detection of the Drug	Refs.
Anesthetics	Procaine	-MWCNT coated glassy carbon electrode	[39]
	Capsaicin	-Carbon nanotube based electrode	[40]
Analgesics	Paracetamol	-Carbon nanotubes and graphene	[41,42,43,44,45,46]
		-Metallic nanoparticles Metal oxide and metal hydroxide nanoparticle composite electrodes	[47,48,49,50]
	Aspirin	-Boron-doped diamond electrode	[51]
		-Alumina coated MWCNT nanocomposite modified glassy carbon electrode	[52]
	Morphine	-Gold nanoparticle and Nafion modified carbon paste electrode	[53]
		-MWCNT and a chitosan modified glassy carbon electrode.	[53]
		-GCE modified with graphene nanosheet	[54]
		-Carbon paste electrode modified with vinylferrocene and MWCNT	[55]
	Tramadol	-Carbon nanotube and Au nanoparticles glassy carbon electrode	[56]
		-Glassy carbon paste modified with Dowex and Au nanoparticles	[57]
	Sumatriptan	-Pyrolytic graphite electrode modified with MWCNT and Ag nano particles	[58]
		-Glassy carbon electrode modified with MWCNT and polypyrrole doped with new coccine	[59]
		-Au nanoparticle and graphene modified glassy carbon electrode	[60]
	Cadeine	-MWCNT modified glassy carbon electrode	[61]
		-SWCNT modified carbon-ceramic electrode	[62]
	Benorilate	-Carbon paste electrode with Ag nanoparticles	[63]
Anti-epileptics	Carbamazepine	-MWCNT on a glassy carbon electrode	[64,65]
		Fullerene-C60-modified glassy carbon electrode	
	Gabapentin	-Ag nanoparticles modified MWCNT	[66,67]
		-Carbon paste electrode modified with nanotubes of nickel oxide was carried out	[68]
	Lamotrigine	-Ag nano modified carbon screen-printed electrode	[69]
Psycholeptics	Buspirone	-DNA-templated Ag nanoparticle placed on a glassy carbon electrode	[70]
	Chlorpromazine	-Glassy carbon electrode modified with a MWCNTs-polyethyleneimine	[71]
		-Carbon paste electrode was modified with cobalt nanoparticles	[72]
	Clozapine	-Carbon paste electrode modified with TiO_2_	[73]
		-Carbon paste electrode modified with polypyrrole that was doped with MWCNTs	[74]
	Risperidone	-Carbon paste electrode modified MWCNTs	[75]
		-Carbon paste electrode coated with MWCNTs and the ionic liquid n-octylpyridinum hexafluorophosphate	[76]
	Thioridazine	-Electrode modified with MWCNT and Co nanoparticles	[77]
		-Carbon paste electrode modified with ZnS nanoparticles	
Psychoanaleptics	Caffeine	-Glassy carbon electrode modified MWCNTs	[78]
		-Pyrolytic graphite electrode	[79]
	Clomipramine	-Glassy carbon electrode that was modified with poly (aminobenzene sulfonic acid) and Pt nano-clusters	[80]
	Desipramine, imipramine and trimipramine	-Glassy carbon paste electrode modified with an Amberlite (XAD2) and TiO_2_ nanoparticles	[81]
		-Boron-doped diamond electrode	[82]
	Trazodone	-Glassy carbon electrode modified MWCNTs	[83]
	Venlafaxine and desvenlafaxine	-GCE modified with a nafion–carbon nanotube composite	[84]
		-Hanging mercury dropping electrode	[85]
		-Mercury film electrode	[86]
Other nervous system drugs	Cinnarizine	-Glassy carbon electrode	[87]
		-Glassy carbon electrode with MWCNTs	[88]
	Dextromethorphan	-Glassy carbon electrode modified with carbon nanotubes and an ionic liquid	[59]
	Naltrexone	-Glassy carbon electrode with Nafion-doped carbon nanoparticles	[89]
		-Glassy carbon electrode with a bilayer of MWCNT and polypyrrole doped with nitrazine yellow	[90]
	Nicotine	-Pencil graphite electrode in the presence of the anionic surfactant	[91]
		-Basal plane pyrolytic graphite electrode modified with layers of MWCNTs	[92]

**Table 4:** Summary of neurotransmitters and their nanomaterial-based detection methods.

Neurotransmitter	Neurotransmitter Function	Detecting Technique	Ref.
**Levodopa and carbidopa**	-Used for the medication of Parkinson’s disease	-MWCNTs and polypyrrole modified glassy carbon electrode doped with tiron	[109]
		-MWCNTs covered graphite electrode that were modified with Au nanoparticles	[110]
		-Carbon paste electrode modified with a bis (nitriloethylidyne)-bis-hydroquinone and carbon nanotube	[111]
		-Carbon nanotube paste electrode modified with an ionic liquid.	[112]
		-Ferrocene-modified carbon nanotube paste electrode	[113]
**Dopamine**	-Plays a huge role in functions of central nervous, hormonal, renal, and cardiovascular systems	-Self assembled carbon nanotubes	[114]
		-A gold nanocluster was incorporated into a glassy carbon electrode modified with 3-amino-5-mercapto-1,2,4 triazole film.	[115]
		-A Pt/reduced graphene oxide modified glassy carbon electrode	[116]
		-LaFeO3 nanoparticles	[117]
		-Nano Au/DNA/nano Au/poly safranine T composite deposited on glassy carbon electrode	[118]
		-CuO nanoparticles	[119]
		-Carbon fiber microbiosensor modified with copper-graphene oxide	[120]
**Epinephrine**	-Regulates heart rate, blood vessel and air passage diameters along with metabolic shifts	-MWCNT-modified edge-plane pyrolytic graphite electrode	[121]
	-Its release plays a big role in the fight-or-flight response of the sympathetic nervous system.	-Functionalized MWCNTs	[122]
**Norepinephrine**	-It is used for treating myocardial infarction hypertension, bronchial asthma and organic heart disease	-Screen printed carbon electrode modified with poly (acrylic acid)-coated MWCNTs	[123]
		-Poly-glycine membrane containing silver nanoparticles	[124]
		-Carbon paste electrode modified with carbon nanotubes and a molybdenum (VI) complex	[125]
**Serotonin**	-Plays a crucial role in the emotional system such as regulation of mood, sleep, vomiting, appetite and sexuality.	-MWCNTs /ionic liquid	[118]
		-Reduced graphene oxide in a porphyrine-modified glassy carbon electrode	[126]
		-Glassy carbon electrode modified with MWCNT/chitosan	[127]
		-Nafion/Ni(OH)_2_ nanoparticles and MWCNTs modified glassy carbon electrode	[82]
